# Th17 cell plasticity towards a T-bet-dependent Th1 phenotype is required for bacterial control in *Staphylococcus aureus* infection

**DOI:** 10.1371/journal.ppat.1010430

**Published:** 2022-04-21

**Authors:** Patricia Bartsch, Christoph Kilian, Malte Hellmig, Hans-Joachim Paust, Alina Borchers, Amirrtavarshni Sivayoganathan, Leon Enk, Yu Zhao, Nikhat Shaikh, Henning Büttner, Milagros N. Wong, Victor G. Puelles, Thorsten Wiech, Richard Flavell, Tobias B. Huber, Jan-Eric Turner, Stefan Bonn, Samuel Huber, Nicola Gagliani, Hans-Willi Mittrücker, Holger Rohde, Ulf Panzer, Christian F. Krebs

**Affiliations:** 1 III. Department of Medicine, Division of Translational Immunology, University Medical Center Hamburg-Eppendorf, Hamburg, Germany; 2 Hamburg Center for Translational Immunology (HCTI), University Medical Center Hamburg-Eppendorf, Hamburg, Germany; 3 Institute of Medical Microbiology, Virology and Hygiene, University Medical Center Hamburg-Eppendorf, Hamburg, Germany; 4 I. Department of Medicine, University Medical Center Hamburg-Eppendorf, Hamburg, Germany; 5 Institute of Medical Systems Biology, Center for Molecular Neurobiology Hamburg (ZMNH), University Medical Center Hamburg-Eppendorf, Hamburg, Germany; 6 III. Department of Medicine, University Medical Center Hamburg-Eppendorf, Hamburg, Germany; 7 Institute of Pathology, University Medical Center Hamburg-Eppendorf, Hamburg, Germany; 8 Department of Immunobiology, Yale University, New Haven, Connecticut, United States of America; 9 Department of General, Visceral and Thoracic Surgery, University Medical Center Hamburg-Eppendorf, Hamburg, Germany; 10 Institute of Immunology, University Medical Center Hamburg-Eppendorf, Hamburg, Germany; Columbia University, UNITED STATES

## Abstract

*Staphylococcus aureus* is frequently detected in patients with sepsis and thus represents a major health burden worldwide. CD4^+^ T helper cells are involved in the immune response to *S*. *aureus* by supporting antibody production and phagocytosis. In particular, Th1 and Th17 cells secreting IFN-γ and IL-17A, are involved in the control of systemic *S*. *aureus* infections in humans and mice.

To investigate the role of T cells in severe *S*. *aureus* infections, we established a mouse sepsis model in which the kidney was identified to be the organ with the highest bacterial load and abundance of Th17 cells. In this model, IL-17A but not IFN-γ was required for bacterial control. Using *Il17aCre* × *R26YFP* mice we could show that Th17 fate cells produce Th17 and Th1 cytokines, indicating a high degree of Th17 cell plasticity. Single cell RNA-sequencing of renal Th17 fate cells uncovered their heterogeneity and identified a cluster with a Th1 expression profile within the Th17 cell population, which was absent in mice with T-bet/*Tbx21*-deficiency in Th17 cells (*Il17aCre x R26eYFP* x *Tbx21-flox*). Blocking Th17 to Th1 transdifferentiation in Th17 fate cells in these mice resulted in increased *S*. *aureus* tissue loads.

In summary, we highlight the impact of Th17 cells in controlling systemic *S*. *aureus* infections and show that T-bet expression by Th17 cells is required for bacterial clearance. While targeting the Th17 cell immune response is an important therapeutic option in autoimmunity, silencing Th17 cells might have detrimental effects in bacterial infections.

## Author summary

*Staphylococcus aureus* is a commensal and opportunistic pathogen that is involved in a variety of diseases such as skin infection, food poisoning, endocarditis or pneumonia and sepsis. In particular, in patients with bacterial sepsis, *S*. *aureus* causes a high mortality. Despite progress in medical treatment in general, the survival rates of *S*. *aureus* sepsis did not improve in the last decades. The interaction between adaptive immune system and this pathogen is a topic of great interest. Infection of mice with *S*. *aureus* revealed the highest bacterial load and abundance of Th17 cells in the kidney. We could show prominent T-bet-dependent transdifferentiation of Th17 cells to highly effective anti-bacterial Th1 phenotypes in the kidney. Thus, T-bet is essential for the Th17 to Th1 transdifferentiation which is required for the control of bacterial infections. Targeting the plasticity of pro-inflammatory T cell subset is a promising therapeutic strategy to silence detrimental T cells in autoimmunity while augmenting anti-bacterial T cells in infection.

## Introduction

Blood stream infections (BSI) and sepsis are still a major health burden, which despite aggressive antimicrobial therapies account for approximately 5 million deaths every year worldwide [1,2]. While a broad range of pathogens are able to cause BSI, Gram-positive pathogen *Staphylococcus aureus* is of particular clinical importance and related to severe courses of bacterial sepsis [3]. *S*. *aureus* is a human pathobiont that, while colonizing the nose of healthy individuals, can cause invasive disease, systemic inflammation and death in vulnerable individuals [4]. Unfortunately, the survival rate of *S*. *aureus* sepsis has not improved in recent decades [5]. Insights into adaptive immune responses hold promise to provide novel clues for understanding the success of *S*. *aureus* as an invasive pathogen.

The immune reaction to invasive infection has been categorized in two states, excessive inflammation and subsequent immune suppression. The first response to an invading pathogen is characterized by a proinflammatory innate immune response that includes activation of the coagulation system, complement system and activation of neutrophils and platelets [6]. *S*. *aureus* infection triggers the production of antimicrobial peptides such as hBD-3 or RNase7 [7] and activation of granulopoiesis. In this context, activated antigen-presenting cells (APCs) produce IL-23 which is essential for polarization and maintenance of Th17 cells [8]. The notion that bacterial virulence factors involved in the resistance of S. *aureus* to the human immune system trigger the activation of different T cell subsets [9,10], indicates that the T cell immune response might play a functional role in bacterial clearance. However, it is important to preserve efficient anti-bacterial subsets while reducing subsets that promote bacterial survival in order to control *S*. *aureus* infections.

*In vitro* differentiation assays revealed that presence of *S*. *aureus* antigens induce the development of IL-17-expressing CD4^+^ T cells (Th17) [11–13], and in the serum of septic shock patients IL-17A levels were elevated [14], indicating that bacterial infections might trigger Th17 immune responses. However, the impact of IL-17A in controlling *S*. *aureus* infection *in vivo* is still controversial [9]. For example, in *S*. *aureus* infection models mice with deficient IL-17 signaling had increased mortality [15] and higher bacterial burden [16,17], while another study showed protection from tissue-injury with systemic *S*. *aureus* infection in IL-17A-deficient mice [18]. IL-17A and F have been reported to be dispensable in mice for reducing the bacterial burden in systemic infection, while mucocutaneus infection is controlled by the IL-17 immune response [8]. These data indicate differences between local tissue-specific and systemic immune responses to *S*. *aureus* infection. Interestingly, we have recently identified a pathogen-induced generation of tissue-resident memory cells of the Th17 cell subset (Trm17) in the kidney [19]. These Trm17 cells reside in the kidney after the infection has been cleared and upon reactivation by inflammatory triggers can aggravate immune-mediated kidney disease.

Several studies from the past decade indicate that T cell subsets cannot be seen as terminally differentiated cells, but that T cell polarization could be defined a temporary condition in a continuum of manifold states. This might hold true particularly for IL-17-producing Th17 cells that display significant plasticity in various models of autoimmune diseases [20–23] and interaction with commensal bacteria in the intestine [24]. However, little is known about plasticity in host defense against pathogens in systemic infection [25].

In this study, a mouse model of acute *S*. *aureus* infection facilitated the investigation of T cell immune responses to bacterial inflammation in the affected tissue, as *S*. *aureus* triggers a prominent Th17 immune response particularly in the kidney compared to other organs. We show that plasticity of renal Th17 cells generates a highly effective Th17 cell subset characterized by a Th1 cell expression profile that drives bacterial clearance in the kidney. These results show that T cell plasticity is an important factor in the host reaction to invading pathogens and highlight a T cell subset with high antibacterial capacities.

## Results

### S. aureus sepsis induces a prominent Th17 immune response in the kidney

**Fig 1:**
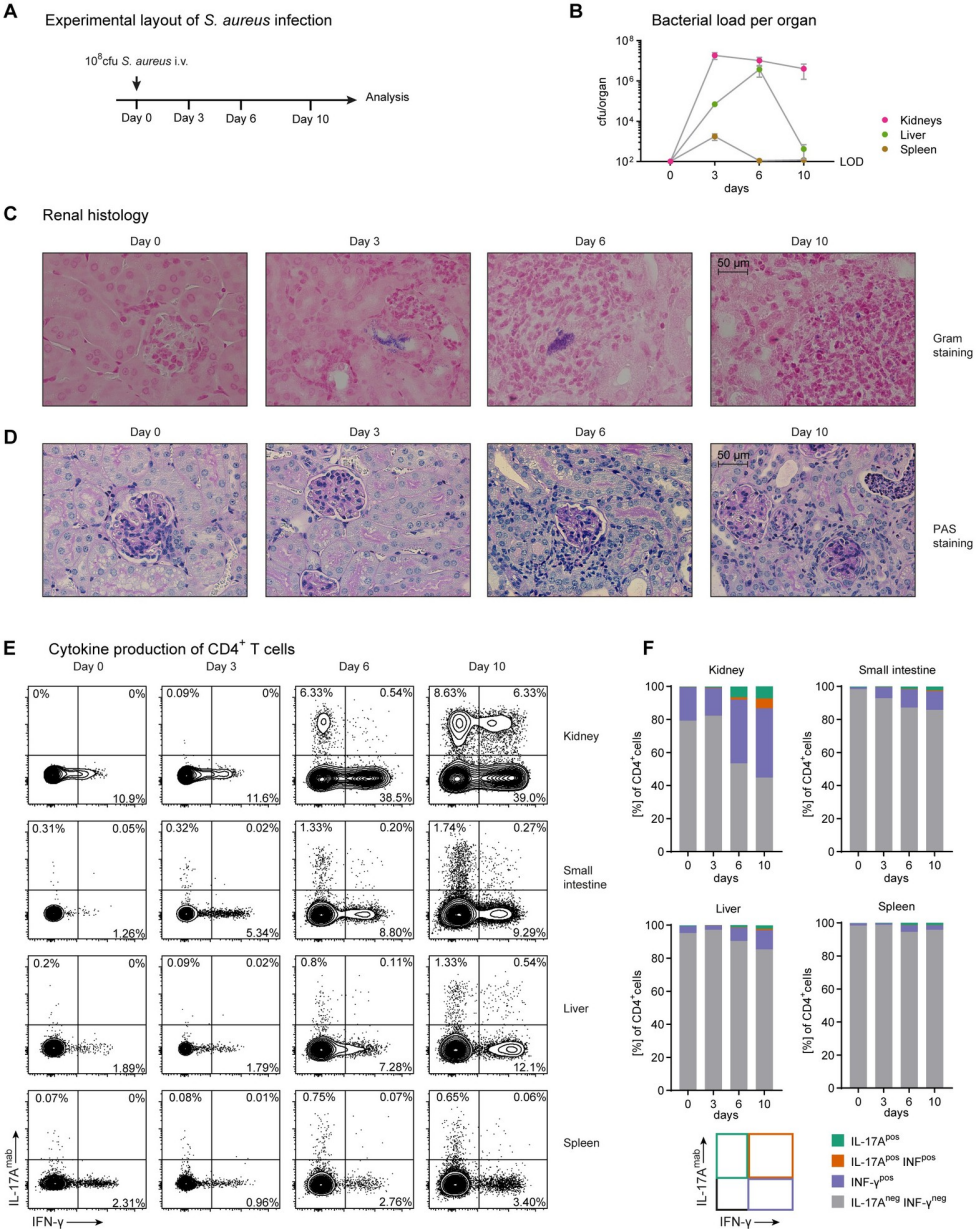
Tissue-specific Th17 cell response in *S*. *aureus* sepsis. (A) Model of *S*. *aureus* infection: injection of 10^8^ cfu into tail vein at day 0 and analysis at indicated time points. (B) Quantification of bacterial load in different organs during *S*. *aureus* infection at indicated time points. (C) Gram staining (D) PAS staining of kidney sections after *S*. *aureus* infection as indicated. (E) Flow cytometry and quantification (F) of IL-17A and IFN-γ producing CD4^+^ T cells in different organs after *S*. *aureus* infection as indicated (each time point represents the data of n = 3–4 mice, representative data from one of two independent experiments).

We have recently described the development of tissue-resident memory T cells with a Th17 polarization state after *S*. *aureus* infection that contribute to immune-mediated glomerular disease. Since the T cell response to *S*. *aureus* in acute infection remains unclear, we investigated the immune response to *S*. *aureus* bloodstream infection over a period of 10 days (Fig 1A). First, we measured the bacterial burden of kidneys, spleen and liver at days 0, 3, 6 and 10 after infection with *S*. *aureus* (Fig 1B) and confirmed previous data that the kidney is the organ with the highest bacterial burden after infection [19]. After identifying the kidney as an important immunological site of acute *S*. *aureus* infection, histological investigation revealed *S*. *aureus* accumulations to be located in the tubulointerstitial area of the kidneys (Fig 1C). Renal pathology is characterized by abscess formation, a hallmark of *S*. *aureus* infection (Fig 1D). Quantification of abscess lesions per kidney section showed an increase in the course of infection (S1A and S1B Fig). This finding was supported by CD3 and GR1 immunohistochemistry of kidney sections for the detection of T cells and neutrophils, respectively (S1C and S1E Fig). These cells accumulated in the course of infection in the tubulointerstitial space as well as in the glomeruli (S1D and S1F Fig). Since glomeruli were affected, we looked for the integrity of the glomerular basement membrane that serves as the urine blood barrier. Importantly, we did not find any immune depositions at the glomerular basement membrane (S1G Fig), arguing against the manifestation of membranoproliferative glomerulonephritis in this model. To characterize the T cell immune response during *S*. *aureus* infection, we analyzed the cytokine production of CD4^+^ T cells from the kidney, small intestine, liver and spleen by intracellular cytokine staining (Fig 1E and 1F). While IL-17A-production was detected only at very low levels in healthy mice, *S*. *aureus* infection resulted in a continuous increase of the IL-17A-producing population over time. This accumulation of IL-17A-producing T cells was most pronounced in the kidney compared to liver, small intestine and spleen. Interestingly, a population of IL-17A and IFN-γ co-producing CD4^+^ T cells emerged in the kidney after infection.

We also investigated additional cell types that could contribute as a local source for IL-17A. γδ T-cells produced IL-17A upon infection with *S*. *aureus*. Interestingly, γδ T cells mainly express IL-17A and do not show a prominent population of IL-17A and IFN-γ co-producing cells (S2A–S2C Fig). The examination of innate lymphoid cells (ILCs) in the kidney revealed a reduction in their relative cell number upon infection and only minor contribution to renal IL-17A (S2D–S2F Fig).

### IL-17A-deficient mice show higher bacterial burden

**Fig 2:**
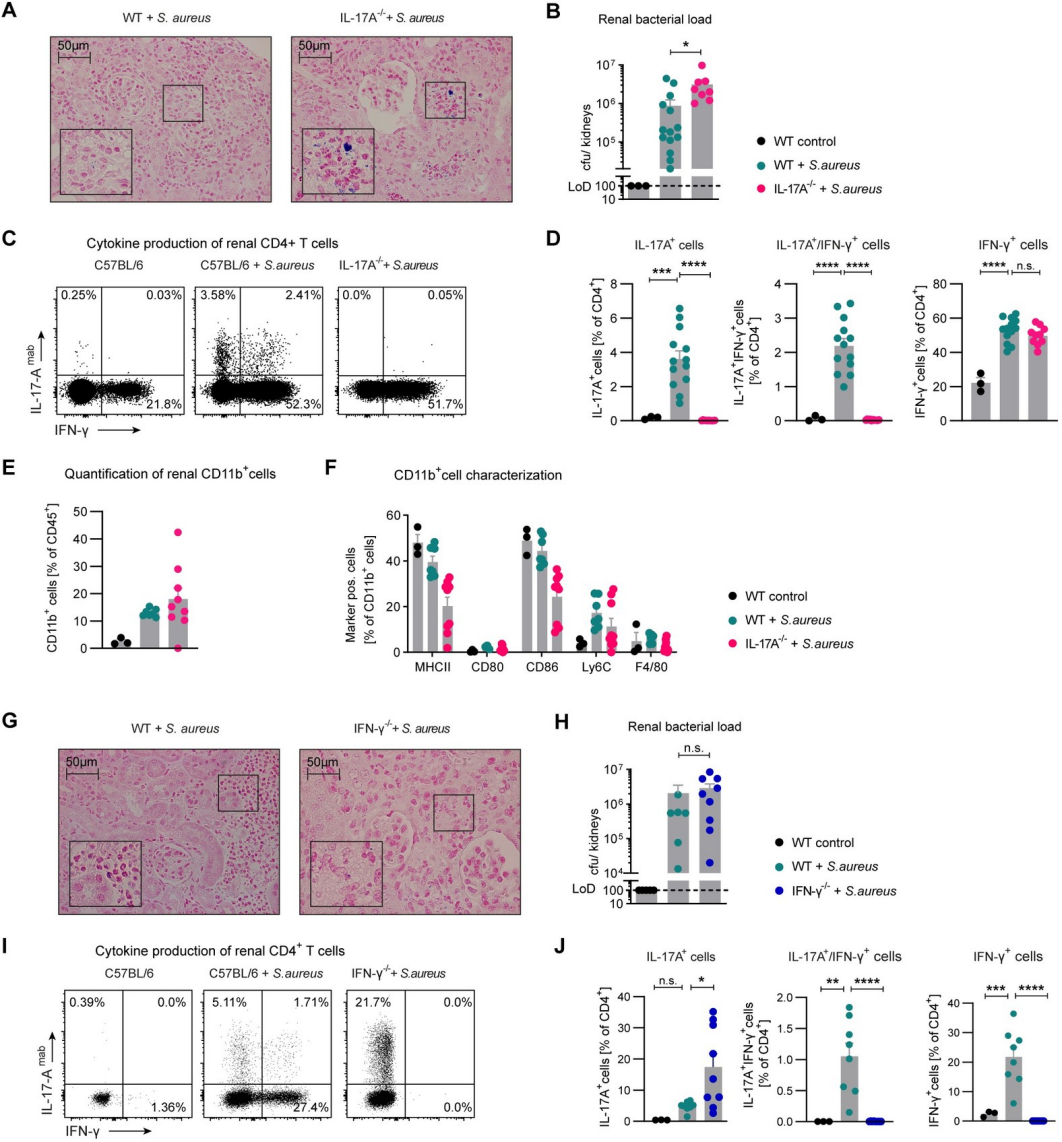
IL-17A-deficient mice show highest bacterial burden in comparison to WT and IFN-γ-deficient mice. (A) Gram staining of kidney sections from C57BL/6 and IL-17A-deficient mice 10 days after *S*. *aureus* infection. (B) Quantification of bacterial load in kidneys as indicated; bars representing mean; individual mice displayed by dots (* *p*<0.05 in Mann-Whitney test). (C and D) Flow cytometry of renal CD4^+^ T cells at day 10 after *S*. *aureus* infection. (E) Quantification of renal CD11b^+^ cells of CD45^+^cells and (F) characterization of renal CD11b^+^ cells at day 10 after *S*. *aureus* infection (representative for one of three independent experiments). (G) Gram staining of kidney sections from C57BL/6 and IFN-γ-deficient mice 10 days after *S*. *aureus* infection. (H) Quantification of bacterial load in kidneys as indicated (* *p*<0.05 in Mann-Whitney test). (I and J) Flow cytometry of renal CD4^+^ T cells of C57BL/6 and IFN-γ-deficient mice 10 days after *S*. *aureus* infection (pooled data from two independent experiments). Bars representing mean ± SEM, individual mice displayed by dots; not significant (n.s.), * p<0.05, ** *p*<0.01, *** *p*<0.001, **** *p*<0.0001 in Dunnett’s multiple comparison one-way ANOVA analysis.

In this model of *S*. *aureus* infection, IL-17A and IFN-γ production by CD4^+^ T cells is very prominent in the kidney. Therefore, we aimed at investigating the influence of IL-17A and IFN-γ on bacterial clearance using gene deficient mice. Interestingly, IL-17A-deficient animals showed increased staining for Gram-positive bacteria (Fig 2A) and had an elevated bacterial burden (Fig 2B) in the kidney compared to wildtype mice. Flow cytometry confirmed the absence of IL-17A^+^ CD4^+^ T cells in IL-17A-deficient mice (Fig 2C and 2D).

Analysis of renal CD11b^+^ cells exhibited a numerical increase in cell numbers in *S*. *aureus* infection (Figs 2E and S2G) but no difference between wildtype or IL-17A-deficient mice. To further characterize CD11b^+^ mononuclear phagocytes, we tested a panel of cell surface markers (Figs 2F and S2H) and identified reduction in MHCII and CD86 expression in IL-17A-deficient animals in the kidney (S2I Fig). CD11b^+^ cells in the liver of infected animals did not display reduction in MHCII and CD86 in IL-17A-deficient mice (S2J–S2N Fig).

In contrast, IFN-γ-deficiency did not influence renal bacteria abundance in histology (Fig 2G) or culture (Fig 2H). Flow cytometry confirmed the absence of IFN-γ^+^ CD4^+^ T cells in the corresponding gene-deficient mice (Fig 2I and 2J) and showed a higher percentage of IL-17A production by CD4^+^ T cells. Based on these data, we concluded that IL-17A contributes to the clearance of bacteria in the kidney while IFN-γ may be dispensable. Furthermore, the Th17 cell amount in IFN-γ-deficient mice is elevated in contrast to WT mice and might be upregulated to combat infection via IL-17A because IFN-γ is missing.

### Renal Th17 cells show high plasticity towards a Th1-like phenotype in S. aureus sepsis

**Fig 3:**
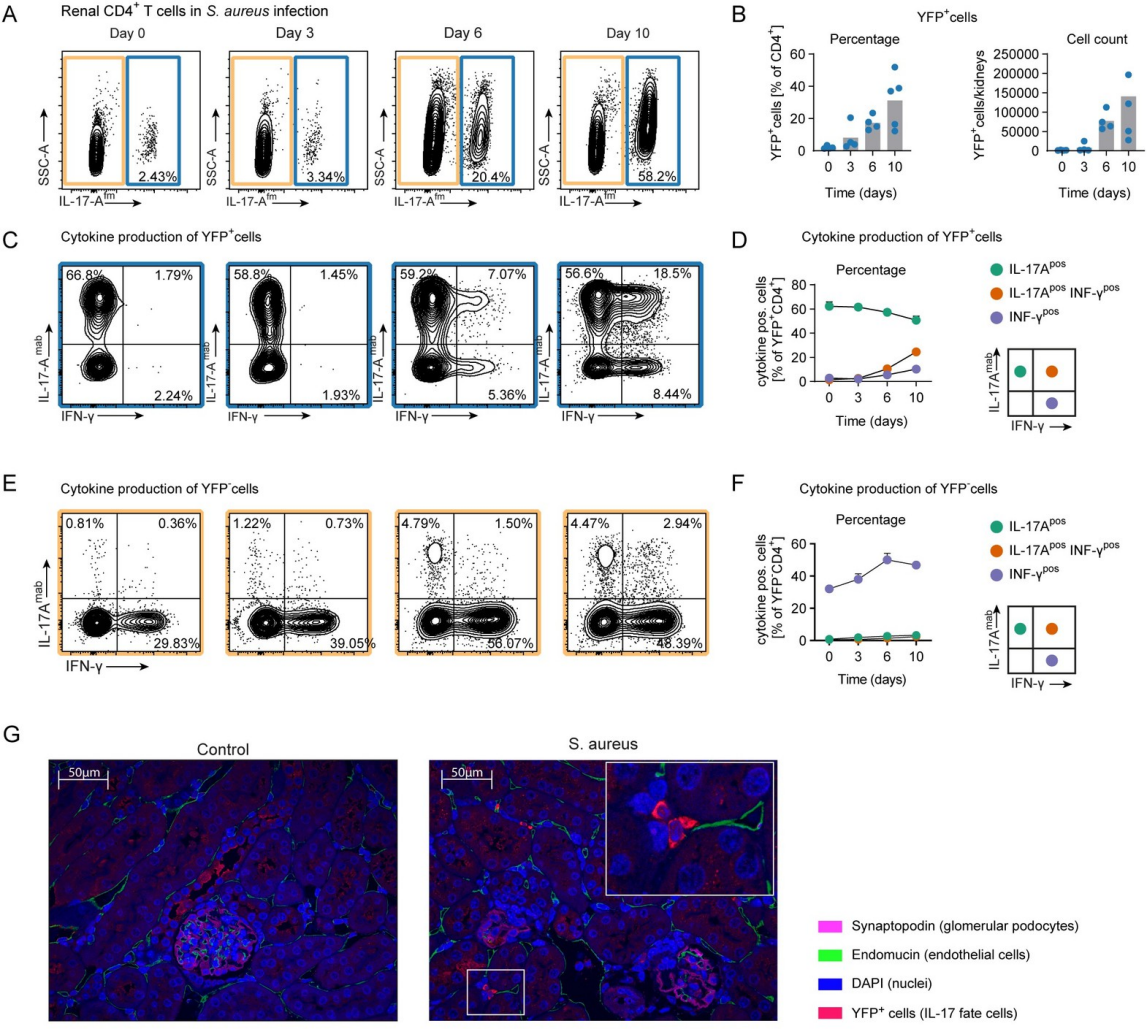
Renal Th17 cells show high plasticity to an Th1-like phenotype in *S*. *aureus* sepsis. (A) Flow cytometry of renal CD4^+^ T cells after *S*. *aureus* infection as indicated from *Il17aCre x R26eYFP* fate reporter mice. (B) Quantification of YFP positive T cells; bars representing mean, individual mice displayed by dots. (C) Flow cytometry and (D) Quantification of cytokine producing of YFP positive T cells. (E) Flow cytometry and (F) Quantification of cytokine producing of YFP negative T cells; (A-F: mean ± SEM, each time point represents the data from n = 4–5 mice, individual mice represented by dots, representative data for one of two independent experiments). (G) Immunofluorescence of YFP positive IL-17A fate cells in kidney sections at day 0 and 10 from *S*. *aureus* infected *Il17aCre x R26eYFP* mice.

In a time course analysis of *S*. *aureus* infection, we identified a population of IL-17A and IFN-γ co-producing CD4^+^ T cells that was not detected in a model of experimental glomerulonephritis with a strong Th17 cell response [26]. Next, we aimed at better characterizing these co-producing cells. To answer the question whether IFN-γ -producing cells originate from Th17 cells, we made use of IL-17A fate reporter mice (*Il17aCre x R26eYFP*) in which T cells that had produced IL-17A are constitutively marked by YFP expression [27] and investigated cytokine production of renal YFP^+^CD4^+^ T cells by flow cytometry. We detected an increasing number of YFP^+^ cells in the course of infection and in particular IL-17A-producing and IFN-γ-producing cells expanded over time (Fig 3A–3D), indicating that Th17 cells become more flexible during infection and show plasticity towards a Th1 cell phenotype. Of note, the analysis of cytokine production of YFP^-^ cells confirmed high IFN-γ production by these cells (Fig 3E and 3F). A combination of indirect immunofluorescence and confocal microscopy revealed the tubulointerstitial localization of YFP^+^ cells in the kidney (Fig 3G).

To investigate the presence of immunoregulatory T cells in *S*. *aureus* sepsis, we used mice with a combined reporter system for Th17 cell fate (*Il17aCre* x *R26eYFP*) and acute cytokine expression (*IL10*^*eGFP*^ *x Il17a*^*Katushka*^ *x Foxp3*^*mRFP*^) (termed fate+) to uncover a possible plasticity of renal Th17 cells into type 1 regulatory T cells (Tr1) defined by IL-10 expression in the absence of Foxp3 [28]. In the kidney, we measured a small increase of Tr1 cells from the Th17 fate (Tr1exTh17 cells) from day 0 to day 6 after infection (S3A Fig). In the small intestine, the numbers of Tr1exTh17 cells were found at lower levels compared to the kidney and their numbers were stable in the course after infection. This discovery highlights the fact that Th17 cells can acquire diverse phenotypes over time.

### Comprehensive gene expression analysis of Th17 cells in the kidney of Il17aCre x R26eYFP mice

**Fig 4:**
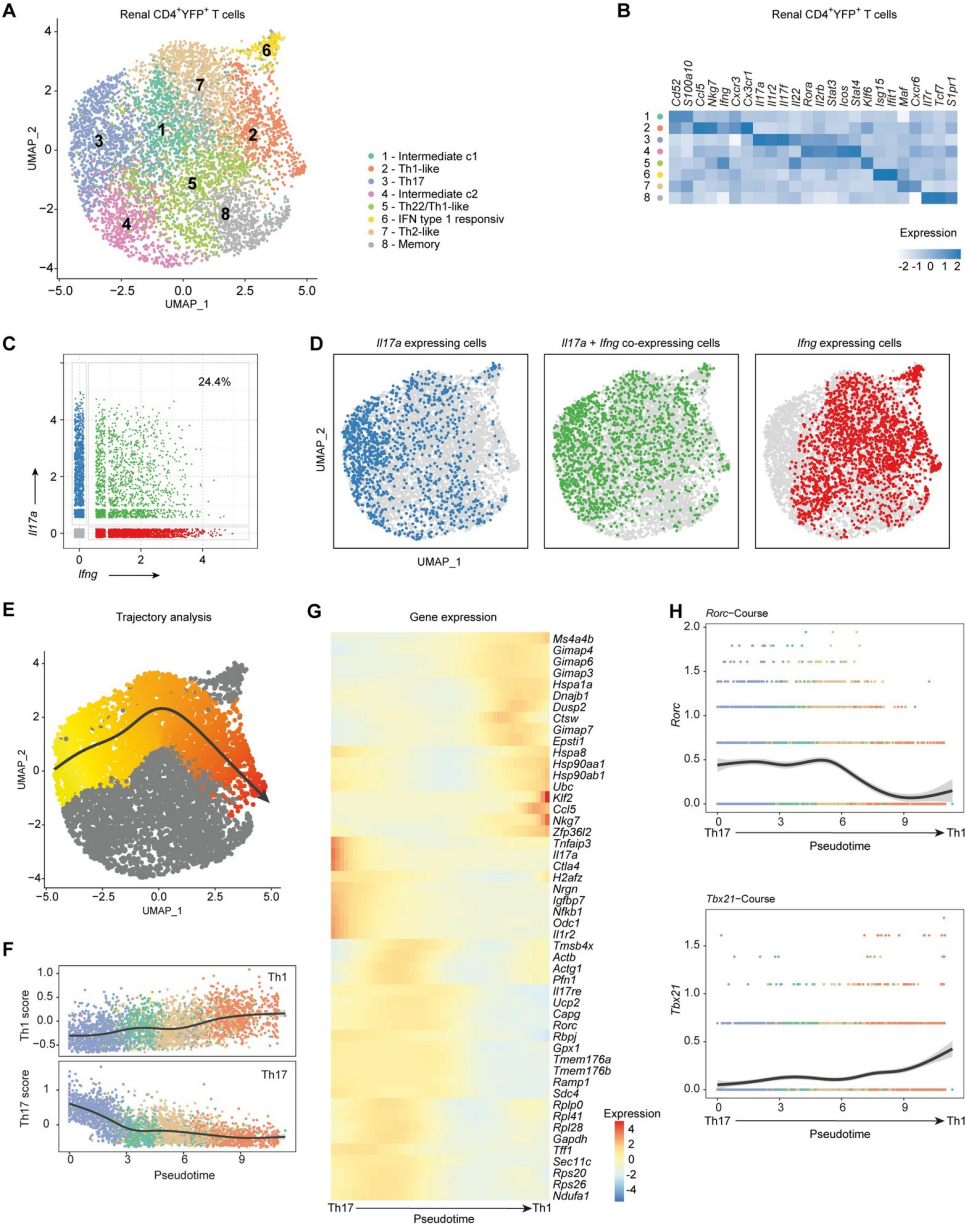
Comprehensive gene expression analysis of Th17 cells in *S*. *aureus* sepsis. (A) UMAP visualization and (B) Differential gene expression of renal YFP^+^ CD4^+^ T cells 10 days after *S*. *aureus* infection of *Il17aCre x R26eYFP* fate reporter mice (pooled cells from n = 5) analyzed by single cell RNA-sequencing. (C) *Il17a* and *Ifng* expression of renal YFP^+^ CD4^+^ T cells 10 days after *S*. *aureus* infection of *Il17aCre x R26eYFP* fate reporter mice (pooled cells from n = 5 mice). (D) Feature plot of *Il17a* and/or *Ifng*-expressing cells. (E) Trajectory of Th17 to Th1-like cells and (F) the Th17 and Th1 score over pseudotime; the line was fit to a generalized additive model. (G) Gene expression of the top 50 genes associated with Th17 to Th1-like trajectory at 50 timestamps over pseudotime, especially of (H) *Rorc* and *Tbx21*.

To investigate the heterogeneity of renal Th17 cells in more detail, we applied single-cell RNA-sequencing (scRNA-seq) to FACS-sorted CD4^+^ YFP^+^ T cells from *S*. *aureus*-infected *Il17aCre* x *R26eYFP* mice. Analysis of scRNA-seq data showed eight clusters within the population of Th17 fate cells in the kidney (Fig 4A), which were annotated according to their gene expression profile (Fig 4B). We identified one cluster (cluster 3) with the common profile of Th17 cells characterized by the expression of *Il17a*, *Il17f*, *Rorc* and *Rora*. Clusters 1 and 4 represented cells in an intermediate cell state, which highly expressed genes linked to bacterial response and inflammation such as *Plac8* (Placenta specific gene 8), *Icos* (Inducible T-cell co-stimulator) and *Tnfaip3* (Tumor necrosis factor alpha-induced protein 3). In cluster 8, we identified genes associated with memory. Interestingly, cells of cluster 5 expressed genes associated with Th1 cells such as *Ifng*, whereas cluster 2 contained cells with high *Ifng* expression in combination with *Cxcr3*. In line with the flow cytometric data of Th17 cells, scRNA-seq revealed IL17-A and IFN-γ co-expressing cells (Fig 4C and 4D). While cluster 3 does not include cells expression only IFN-γ, clusters 2 and 5 show reduced IL-17A positive cells.

To analyze the trajectories of the Th17 cells, we defined cluster 3 with the highest *Il17a*-expression as starting point of differentiation and obtained four different curves by slingshot analysis (S3B Fig). The trajectory from Th17 to Th1-like cells displayed an increasing Th1 score and a decreasing Th17 score which was among other genes based on the expression of the transcription factors *Rorc* and *Tbx21*, respectively (Fig 4E–4G). Looking at the trajectory of Th17 cells towards Th1-like cells, the expression of T-bet increased with pseudotime (Fig 4H). Together, these data indicate the plasticity of bona fide Th17 cells to Th1 phenotypes in *S*. *aureus* infection.

### T-bet-deficiency affects Th17 to Th1 transdifferentiation in infected kidneys

**Fig 5:**
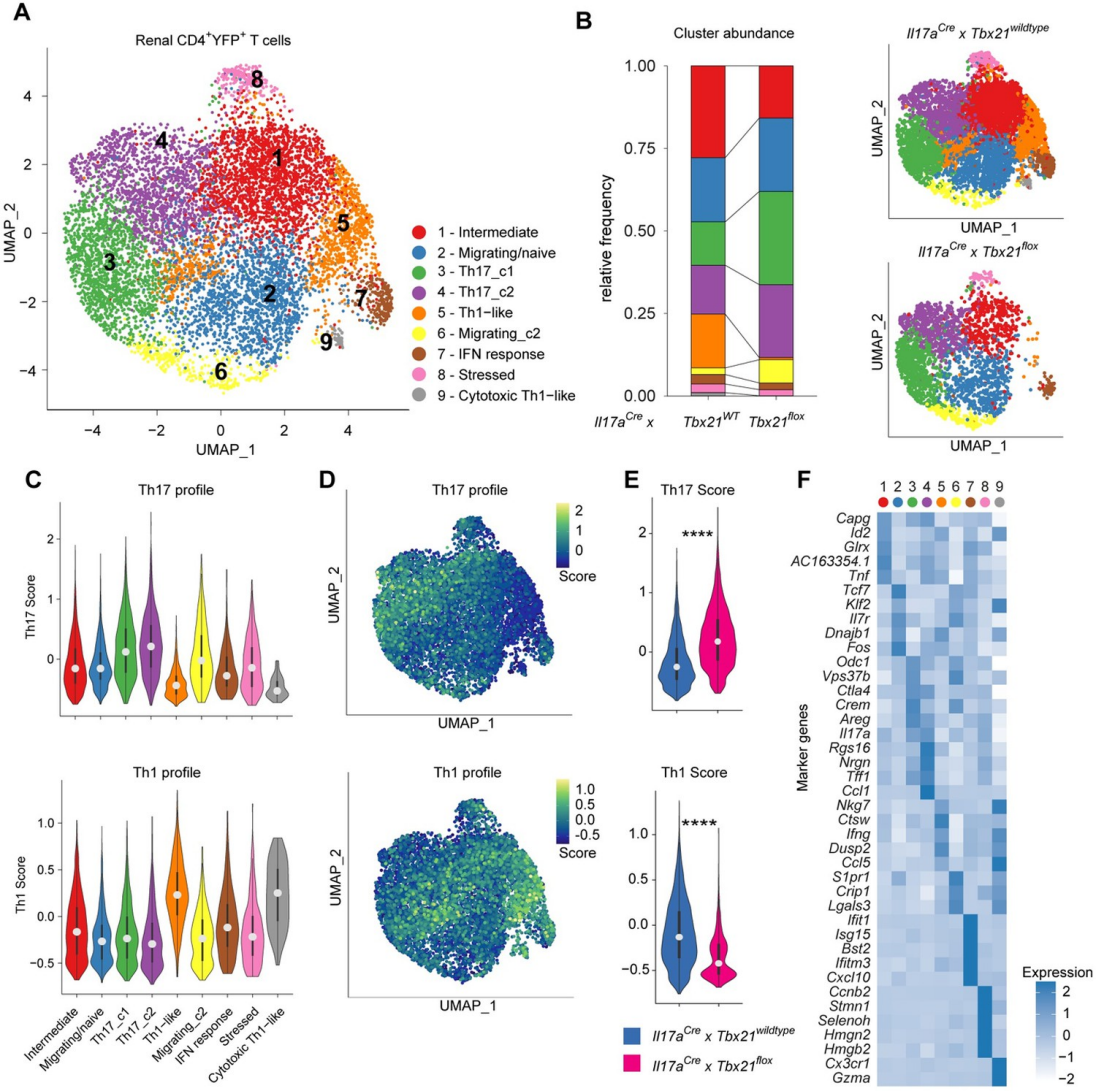
T-bet-deficiency affects Th17 plasticity to Th1-like ex Th17 cells in infected kidneys. (A) UMAP dimensional reduction and (B) Cluster abundance of renal YFP^+^ CD4^+^ T cells 10 days after *S*. *aureus* infection of *Il17aCre x R26eYFP x Tbx21-wildtype* (cells pooled from n = 7 mice) *and Il17aCre x R26eYFP x Tbx21-flox* (cells pooled from n = 6 mice) mice analyzed by single cell RNA-sequencing. (C) Th17 and Th1 scores in the clusters as indicated. (D) Comparison and (E) Quantification of Th17 and Th1 scores in cells from *Tbx21-flox* and *Tbx21-wildtype* mice; Wilcoxon test, two sided, **** p<0.0001. (F) Differential gene expression of the top five genes most specific for each cluster of renal YFP^+^ CD4^+^ T cells 10 days after *S*. *aureus* infection.

*Tbx21*/T-bet was upregulated in Th17 fate cells that transdifferentiated into Th1 phenotypes. To evaluate the impact of T-bet on Th17 cell transdifferentiation, we made use of *S*. *aureus*-infected *Il17aCre x R26eYFP* x *Tbx21-flox* mice. In this mouse model, Th17 cells and their progeny acquire permanent T-bet/*Tbx21*-deficiency. To compare the transcriptional profiles of Th17 with and without T-bet, we performed scRNA-seq analysis of FACS-sorted CD4^+^ YFP^+^ T cells from *Il17aCre x R26eYFP* x *Tbx21-flox* mice at day 10 after *S*. *aureus* infection. Integrating the scRNA-seq data from *Tbx21-*wildtype and *Tbx21*-deficient Th17 cells revealed nine different clusters that were annotated according to their gene expression (Fig 5A). One cluster of Th1-like cells was almost absent in *T-bet*-deficient Th17 cells from *Il17aCre x R26eYFP* x *Tbx21-flox* mice (Fig 5B). Gene expression analyses revealed Th17 associated genes in clusters 3 (Th17_c1) and 4 (Th17_c2) and Th1 associated genes in cluster 5 (Th1-like) and 9 (cytotoxic Th1-like) (Fig 5C and 5D). In addition, Th17 fate cells from *Il17aCre* x *R26eYFP* mice showed a higher Th1 score and Th17 cells from *Il17aCre x R26eYFP* x *Tbx21-flox* infected mice showed a higher Th17 score (Fig 5E).

Th17 cells with T-bet-deficiency might show an arrest in their transdifferentiation to Th1-like cells. Trajectory analyses using the slingshot algorithm exhibited a trajectory line from cluster 3 (high Th17 gene profile) to cluster 5 (high Th1 gene profile), which is absent in Tbx21-deficient cells (S3C Fig). *IFNg* but also *Nkg7* and *Ctsw* are among the top genes most exclusively expressed by these cells (Fig 5F). Taken together, this data supports the notion that *Tbx21*/T-bet is essential for transdifferentiation of Th17 cells in *S*. *aureus* infection to Th1 like cells.

### Th1-exTh17 cells drive bacterial clearance in the kidney

**Fig 6:**
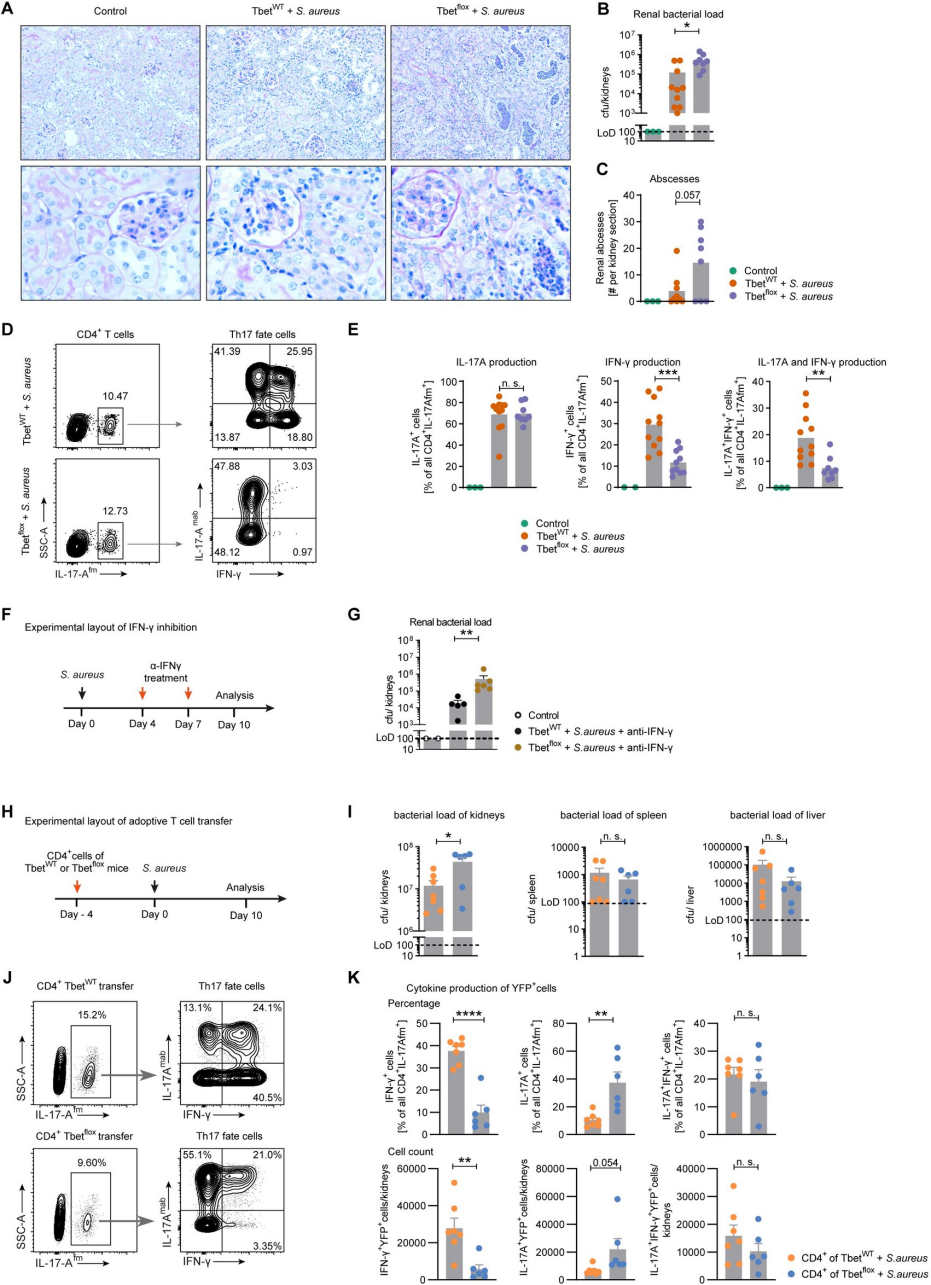
*Tbx21* expressing renal Th17 cells drive bacterial clearance in the kidney. (A) Abscess formation in PAS staining of kidney sections in control mice and 10 days after *S*. *aureus* infection in Th17 fate reporter mice (*Il17aCre x R26eYFP*) and T-bet-deficient fate reporter mice (*Il17aCre x R26eYFP* x *Tbx21-flox*). (B) Quantification of abscesses per stained kidney section 10 days after *S*. *aureus* infection; bars representing mean, individual mice displayed by dots in Dunnett’s multiple comparison one-way ANOVA analysis. (C) Quantification of bacterial load 10 days after *S*. *aureus* infection; bars representing mean individual mice displayed by dots, * p<0.05 in Dunnett’s multiple comparison one-way ANOVA analysis. (D) Flow cytometry of renal YFP^+^ CD4^+^ T cells 10 days after *S*. *aureus* infection as indicated and (E) Quantification of cytokine expression; bars representing mean, individual mice displayed by dots, not significant (n.s.), *p<0.05, ** *p*<0.01, *** *p*<0.001 in Dunnett’s multiple comparison one-way ANOVA analysis (representative for one of two independent experiments). (F) Th17 fate reporter mice (*Il17aCre x R26eYFP*) and T-bet-deficient fate reporter mice (*Il17aCre x R26eYFP* x *Tbx21-flox*) were treated with neutralizing anti-IFN-γ antibody as indicated. (G) Quantification of bacterial load at day 10 after *S*. *aureus* infection (** p<0.01 in Dunnett’s multiple comparison one-way ANOVA analysis. (H) *Rag1*^-/-^ mice were injected i.v. with 5x10^5^ CD4^+^ cells from *Il17aCre x R26eYFP* or *Il17aCre x R26eYFP* x *Tbx21-flox* mice before *S*. *aureus* infection. (I) Quantification of bacterial load at day 10 after after *S*. *aureus* infection in kidney, liver and spleen. (J) Flow cytometry of renal YFP^+^ CD4^+^ T cells and (K) Quantification of cytokine expression. Not significant (n.s.), * p<0.05, ** *p*<0.01, **** *p*<0.0001 in two-tailed unpaired t-test (pooled data from two independent experiments). Bars representing mean, individual mice displayed by dots.

After identifying a population of T-bet-dependent Th17 fate cells by scRNA-seq, we aimed at understanding the role of these cells in *S*. *aureus* infection and in particular their role in the control of the pathogen. After infection with *S*. *aureus*, an extended level of bacteria and abscess formation in the kidney was found in mice with T-bet-deficiency in Th17 fate cells at day 10 after infection compared to T-bet/*Tbx21*-wildtype mice (Fig 6A–6C).

To further evaluate the interleukin expression of Th17 fate cells, we performed flow cytometry of T cells from the kidney after infection. This analysis verified similar IL-17A production of Th17 fate cells from *Il17aCre x R26eYFP* mice and *Il17aCre x R26eYFP x Tbx21-flox* mice. In contrast, levels of IFN-γ producing cells in *Il17aCre x R26eYFP x Tbx21-flox* mice were reduced compared to wildtype mice. This affected both IL-17A^+^/IFN-γ^+^ and IL-17A^neg^/IFN-γ^+^ cells (Fig 6D and 6E). Of note, IFN-γ production by YFP^neg^ CD4^+^ T cells was not altered (S4A and S4B Fig).

To investigate if Th1exTh17 cells exert their antibacterial properties by IFN-γ, we neutralized this cytokine using a monoclonal antibody. Anti-IFN-γ treatment at day 4 and 7 during *S*. *aureus* infection (Fig 6F) of *Il17aCre x R26eYFP* mice and *Il17aCre x R26eYFP x Tbx21-flox* mice did not reverse the phenotype of higher bacterial load in mice with T-bet-deficient Th17 fate cells (Fig 6G). Flow cytometry of YFP^pos^ CD4^+^ T cells displays reduction of IFN-γ production in T-bet deficient mice (S4C and S4D Fig) and support our observations in IFN-γ deficient mice (see Fig 2G and 2H). While this data indicates that IFN-γ is dispensable for the elimination of renal *S*. *aureus*, it underscores the impact of a T-bet-dependent anti-bacterial Th17 cell subset.

In addition to CD4^+^ T cells, we had identified also γδ T cells to contribute to renal IL-17A in *S*. *aureus* infection (S2A–S2C and S4E and S4F Figs). Since we could not exclude that T-bet positive γδ T cells are responsible for bacterial clearance, we performed adoptive CD4^+^ T cell transfer from either *Il17aCre x R26eYFP* mice or *Il17aCre x R26eYFP x Tbx21-flox* mice into RAG^-/-^ mice (Fig 6H). Our analysis shows higher bacterial load in the kidney of mice transferred with *Tbx21-flox* cells. In contrast, there was no difference between the two groups in bacteria isolated from liver and spleen (Fig 6I). Note that the kidney yielded the highest bacterial numbers also in the T cell transfer model.

While the expression of IL-17A was higher in T-bet/*Tbx21-flox* CD4^+^ cells and IFN-γ was increased in T-bet-competent CD4^+^ cells, the co-expression of IL-17A and IFN-γ was not different between these groups (Fig 6J and 6K). Characterization of renal CD11b^+^ cells did not show major differences (S4G and S4H Fig). Taken together, our data support the notion that T-bet expressing cells of the Th17 fate are highly efficient in supporting the elimination of *S*. *aureus* in the kidney.

## Discussion

The adaptive immune response to bacterial challenges remains incompletely understood. In this study, we identify a key role of Th17 cells during bacterial clearance in the kidney. In particular, we found that T-bet/*Tbx21*-expression in Th17 cells gives rise to a highly effective anti-bacterial T cell subset.

Comparative analysis of different tissues revealed a robust T cell response particularly in the kidney of mice after *S*. *aureus* infection. This is in line with a recent publication, in which we identified the highest bacterial titers after systemic *S*. *aureus* infection in the kidney [19]. In the present study, we investigated the early adaptive immune response to *S*. *aureus* infection and found IL-17A-producing CD4^+^ T cells to be highly abundant in the kidney. It is well established that *in vitro* co-culture of *S*. *aureus* antigens with immune cells drives a robust Th17 cell response [11,29]. In this study, we provide evidence that Th17 cells indeed play a major role in controlling *S*. *aureus* infection in living animals. This is in line with a report indicating that T cells contribute to bacterial clearance in the kidney [30]. Furthermore, neutralizing IL-17A in local *S*. *aureus* infection resulted in larger abscesses in the skin [31]. Our data extents these results and indicates that Th17 cells contribute to bacterial clearance in the kidney.

The concept of terminally differentiated T cell subsets has been challenged in the past decade by studies showing transdifferentiation of T cells, including the plasticity of Th17 cells into Th1 [32,33] or Tr1 [28] phenotypes. A high degree of Th17 cell plasticity has been demonstrated in several mouse models of autoimmune diseases such as experimental autoimmune encephalomyelitis, a model of multiple sclerosis [27], and in RB high colitis, a model of inflammatory bowel disease [34] using adoptive cell transfer of purified T cell subsets or fluorescent fate reporter mice. In contrast, in the murine kidney, we have seen very limited Th17 cell plasticity in mouse models of immune-mediated renal disease [26]. The findings of varying degrees of Th17 cells plasticity in different tissues and under different inflammatory triggers suggest that T cell plasticity strongly depends on the local micro-environment. In this study, we observed a high level of Th17 cell plasticity in the kidney after *S*. *aureus* infection based on intracellular cytokine staining. By employing scRNA-seq of Th17 cells, we obtained the full picture of Th17 heterogeneity in the kidney in bacterial infection. Using RNA-slingshot analysis, we identified a trajectory from IL-17A expressing cells to Th1-like cells with expression of *Ifng* and *Cxcr3*. The potential of Th17 cells to develop into Th1 phenotypes is in line with previous publications from animal models of autoimmune diseases. Specifically, Brucklacher-Waldert *et al*. have investigated *Tbx21*-deletion in Th17 cells [35]. In line with this publication, T-bet-deficiency in ex-Th17 cells hinders the development of IFN-γ expressing cells, but IL-17A and IFN-γ co-producing cells still emerge. However, while *Helicobacter hepaticus* infection in the intestine is not altered by T-bet-deficiency in ex-Th17, our data in *S*. *aureus* infection shows T-bet-dependent anti-bacterial function of Th1 phenotypes that derive from Th17 cells.

The function of T-bet in T cells might be depending on the local micro-environment in the tissue. In models for immune-mediated diseases, Th17 cells that produce T-bet-dependent IFN-γ have been shown to be proinflammatory in the central nervous system [36] but T-bet expression in Th17 cells can also modify intestinal inflammation by regulating IL-23 receptor [37]. Our results add to this knowledge by showing that proinflammatory cells can have protective properties in bacterial infection in the kidney, while clearance of extrarenal bacteria does not seem to be dependent on T-bet expression by Th17 cells. Our scRNA data indicate that cells from cluster 5 in Fig 5A have a specific pathogen related function to clear infections with *S*. *aureus*. Although IFN-γ is upregulated during infection and particularly expressed by these transdifferentiated Th1 cells, this cytokine alone seems not to be the key factor of bacterial clearance, since neither *INFg*^-/-^ mice have higher bacterial burden after infection nor does anti-IFN-γ treatment reverse the phenotype in conditional T-bet-deficiency in Th17 cells. It is reasonable to speculate that not one single cytokine of transdifferentiated Th1 cells is responsible for the function but that a certain anti-bacterial profile cumulatively confers these effects. Future studies are required to uncover the precise effector functions of these protective anti-bacterial transdifferentiated Th1 cells.

Plasticity is supported by a publication showing that adoptive transfer of Th17 polarized cells into TCR αβ-deficient mice resulted in increasing numbers of IFN-γ expressing cells [38]. However, based on the design of this study, it was not possible to distinguish Th17 cell plasticity from expansion of Th1 cells that were co-transferred. Our approach using Th17 fate reporter mice shows plasticity of Th17 cells by cytokine profile analysis and single cell RNA-sequencing. Moreover, we show the functional relevance of this transdifferentiation by using *Tbx21*^flox^ animals in combination with the *Il17a-Cre* mice in *S*. *aureus* elimination and highlight *Tbx21*-expressing ex-Th17 cells as an important T cell subset that comprises highly efficient anti-bacterial properties.

In conclusion, our study provides experimental data that supports the role of Th17 cells in the immune response to *S*. *aureus* infection in mice. We have uncovered a population of effector T cells in the kidney with expression of *Tbx21* derived from Th17 cells that is highly effective in bacterial clearance. These data indicate, that therapeutic depletion of Th17 cells or neutralizing their cytokines in settings of autoimmune diseases [39] might have detrimental effects in systemic infections. Further studies are needed for a better understanding of the tissue-specific immune response to advocate bacterial clearance by supporting the T cell mediated immune response in the local micro-environment in order to reduce associated high mortality as well as the resulting tissue damage.

## Materials and methods

### Ethics statement

All animal experiments were conducted according to the National Institutes of Health Guide for the Care and Use of Laboratory Animals as well as the German law for the welfare of animals. All animal experiments were approved by local authorities (BGV Hamburg, G35/16 and G82/19).

### Mice

All experiments were conducted with age-matched (6 to 10 weeks old) mice on C57BL/6 background raised in specific pathogen free conditions at the animal facility of the University Medical Center Hamburg-Eppendorf. The following transgenic mouse strains were used: *Il17a*^-/-^mice [40], *Il17aCre* [27], *R26eYFP* [27], IL-17fate+ (*Foxp3*^*RFP*^ x *LIL-10*^*eGFP*^ x *IL-17A*^*Kat*^ x *Il17aCre x R26eYFP* mice), [28,41,42]. *Ifng*^-/-^ mice were purchased from the Jackson Laboratory (Bar Harbor, ME) and *Tbx21*^fl/fl^ were provided by Steven L. Reiner (Columbia University, New York, NY) [43].

### S. aureus sepsis

Bacteria (*S*. *aureus* strain SH1000) were homogenized by ultrasound and injected in tail vein of the mice in 100 μl PBS (Gibco by Life Technologies, Karlsbad, California). Bacteria were injected at indicated concentration (cfu/μL). For quantification of renal bacteria, kidneys were dissected and incubated for 40 min at 37°C in RPMI1640 medium (including 0.01 M HEPES; 0.5 mL filtered FBS and 0.4 μg/ mL DNase and 8 μg/ mL collagenase D). GentleMACS (Miltenyi Biotec, Bergisch Gladbach, Germany) and sonication for 60 seconds using a Bandelin Sonorex Super RK103 were used for homogenization.

For quantification of hepatic or splenic bacteria these organs were passed through 70 μm nylon mesh (Corning, Corning, New York) and resuspended in PBS before sonification. The samples were incubated for 24 h at 37°C on LB agar plates and colonies were counted. *S*. *aureus* was verified by MALDI-TOF.

### Anti-IFN-γ treatment and CD4^+^isolation and transfer

For anti-IFN-γ, mice were treated on day 4 and 7 after *S*. *aureus* infection with 500 μg/mouse i.p. α-Interferon-γ antibody XMG1.2. For T cell transfer, Leucocytes were isolated as described above from spleen of donor mice. CD4^+^ isolation was performed with the CD4^+^ T Cell Isolation Kit, mouse (Miltenyi Biotec) with manual cell separation by LS columns and MACS separator. 5x10^5^ CD4^+^ cells were injected intravenously per mouse. After 3 days, mice were infected with *S*. *aureus* as described above.

### Leukocyte isolation

Kidneys were dissected and incubated for 40 min at 37°C in RPMI medium (including 0.01 M HEPES; 0.5 mL filtered FBS and 0.4μg/ mL DNase and 8 μg/ mL collagenase D). The samples were homogenized using a gentleMACS (Miltenyi Biotec). Lymphocytes were enriched by Percoll gradient (37% in PBS). Spleen tissue was passed through 70 μm nylon mesh (Corning, Corning, New York) before erythrocyte lysis (17 mM Tris-HCl (pH 7.6) and 144 mM ammonium chloride). Cells were washed and given through a 40 μm nylon mesh (Corning, Corning, New York) with HBSS. The vitality of the cells was measured by staining with Trypan blue solution 1:1 (0.4% Sigma- Aldrich, St. Louis, Missouri). Liver tissue was passed through a 100 μm nylon mesh (Corning, Corning, New York) in HBSS. Lymphocytes were enriched using a 37% Percoll gradient including 20 Units heparin and erythrocyte lysis buffer.

Small intestine was washed with PBS after extraction and shaken in PBS including 2 mM dithiothreitol. The samples were incubated for 30 min at 37°C at 200 rpm in PBS (10% FBS, 0.22 mM sodium pyruvate, 4.4 M HEPES, 2.2 M EDTA, 11 μg streptomycin, 11 Units penicillin and 0.011 mg/ mL polymyxin B). The tissue was passed through a 100 μm nylon mesh (Corning, Corning, New York) and incubated for 45 min at 37°C in RPMI medium (including 0.01 M HEPES; 0.05 μg/ mL streptomycin; 0.05 Units penicillin; 10% filtered FBS, 0.4 μg/ mL DNase and 8 μg/ mL collagenase D) in slow rotation in the MACS-Mix Tube Rotator (Miltenyi Biotec). After homogenization by gentleMACS (Miltenyi Biotec), the tissue was solubilized through a 70 μm nylon mesh (Corning, Corning, New York) in RPMI1640 medium (including 0.01 M HEPES; 0.05 μg/ mL streptomycin; 0.05 Units penicillin and 0.5 mL FBS). Lymphocytes were enriched using a 37% Percoll gradient.

For the analysis of mononuclear phagocytes, extracellular staining with CD45 AF700 (1:80) 30-F1, CD3 BV785 (1:250) 145-2C11, CD11c V450 (1:100) HL3, CD11b PE-Cy7 (1:500) M1/70, MHC2 BV510 (1:160) M5/114.15.2, F4/80 APC (1:150) BM8, Ly6g PerCP (1:100) 1A8, Ly6c APC-H7 (1:200) HK1.4, CD86 BV650 (1:400) GL-1 and CD80 BV605 (1:300) 16-10A1 for 20–25 min at room temperature. LIVE/DEAD Fixable Red Dead Cell Stain Kit (1:3000) in PBS (Live Technologies, Karlsbad, California) was used for labelling of dead cells for 2 min at room temperature before erythrocyte lysis (17 mM Tris-HCl (pH 7.6) and 144 mM ammonium chloride). Liver tissue was passed through a 100 μm and 40 μm nylon mesh (Corning, New York) in PBS and further processed like kidney tissue.

### Flow cytometry

For stimulation, leucocytes were incubated in X-Vivo 20 Serum-free Hematopoietic Cell Medium (Lonza, Basel, Switzerland) supplemented with β-mercaptoethanol, brefeldin A, PMA and ionomycin for 3–4 h at 37°C at 5% CO_2_ (Heraeus instruments, Hanau, Germany). To prevent unspecific binding, leucocytes were incubated in blocking solution (MACS buffer containing 10% mouse serum) for 5 min at 4°C. Next, samples were stained with the following antibodies: CD45 PercP (1:100) 30-F1, CD4 BV605 (1:600) RM4-5, CD8 BV 785 (1:1000) 53–6.7, CD3 AF700 (1:50) 145-2C11, γδTCR BV510 (1:100) eBio GL3 for T cell staining for 20–25 min at 4°C. To define ILCS and other leucocytes subsets the following antibodies were used: CD45 PerCP (1:200) 30-F11, IL-7Rα-CD127 PE-Cy7 (1:100) A7R34, Thy1.2-CD90.2 BV510 (1:200) 30-H12, CD4 BV650 (1:100) RM4-5, CD8 BV785- (1:200) 53–6.7, CD3 AF700 (1:50) 17A2, gdTCR FITC (1:100) GL3 and a combination of lineage markers (Lin-BV605), including CD5 (1:200) 53–7.3, CD11b (1:1000) M1/70, CD11c (1:200) HL-3, CD19 (1:400) 6D5, CD49b (1:800) HMα2, TCR-β (1:400) H57-597, GR-1 (1:400) RB6-8C5, NK1.1 (1:200) PK136 and Ter119 (1:200) Ter119. LIVE/DEAD Fixable Near-IR Dead Cell Stain (1:1000) in PBS (Live Technologies, Karlsbad, Carlifornia) or LIVE/DEAD Fixable Read Dead Stain Kit (Invitrogen) was used for labelling of dead cells for 25 min at 4°C. For intracellular staining, of T cells the cells were fixed with 3,7% formalin for 17 min at 4°C and of ILCs for 30 min at room temperature. Afterwards leucocytes were incubated with 0.1% IGEPAL (Sigma- Aldrich, St. Louis, Missouri) in MACS buffer for 4 min at 4°C and stained with the following antibodies: IL-17A BD (1:1000) TC11-18H10, IFN-γ eBio (1:1000) XMG1.2 for 30 min at 4°C for intracellular staining of T cells. In case of ILC staining, cells were stained in Perm Wash buffer (eBioscience) supplemented with a combination of intranuclear and intracellular markers with fluorochrome-coupled antibodies against ROR-γt APC (1:300) Q31-378, IL-17A PE (1:200) TC11-18H10 and IFNγ BV711 (1:200) XMG1.2 and stained overnight at 4°C.

For assessment of leucocytes counts in the kidney or liver by flow cytometry, cells were mixed with CountBright absolute counting beads (Life Technologies, Karlsbad, Carlifornia) and CD45 PerCP mAb (1:200). The individual cell frequencies were adjusted to the CD45 cell count. Flow cytometry was performed with a BD FACS LSRII or a LSR II Fortessa (BD Biosciences, Franklin Lakes, New Jersey) and data was analyzed with FlowJo (Tree Star).

### Single cell RNA-isolation and library construction

Renal CD45^+^CD3^+^CD4^+^YFP^+^ cells from *Il17aCre x R26eYFP* mice were sorted using an Aria Fusion cytometer (BD Biosciences, Franklin Lakes, New Jersey) and collected in MACS buffer containing 2% FCS. These FACS-sorted cells underwent droplet-based single cell analysis and transcriptome library preparation using the Chromium Single Cell 3´ Reagent Kits v2, Chromium Single Cell 3´Library & Gel Bead Kit v2, Chromium Single Cell A Chip Kit and Chromium i7 Multiplex Kit according to the manufacture´s protocols (10x Genomics, Pleasanton, California).

### Pre-processing, dimensional reduction and cluster of single-cell RNA sequencing data

The processing of the single-cell data was done using the R software version 4.0.0 (2020-04-24). For the sake of reproducibility, we set the global seed to 0. If not mentioned otherwise, we ran the methods with default parameters. The R-Package Seurat (version 4.0.1) was used for pre-processing, dimensional reduction and cluster identification.

For the analysis of cells from IL-17A fate reporter mice, we removed cells according to the number of different genes expressed per cell (nGenes) (<673 (10th quantile) and >3924 (95th quantile)) and their percentage of mitochondrial genes (>8% mitochondrial genes). Next, the data was normalized and mitochondrial confounding effects diminished by applying Seurat’s SCTransform [44] and adjusting for the percentage of mitochondrial genes. For dimensional reduction, principal components (PC) were calculated using the method Run PCA (features = Variable Features). We selected the PCs 1–40 to construct the Shared Nearest Neighbor Graph (Method: ‘Find Neighbours’) and the UMAP-Space (Method: ‘Run UMAP’). The clusters were calculated by the Louvain algorithm (Find Clusters (resolution = 0.6)). While identifying the resulting clusters, we removed two clusters with high scores for cycle phases S, G2, M and another cluster with low alpha/beta TCR expression.

For comparative analysis of wildtype and Tbx21-deficient cells, we removed cells according to the number of different genes expressed per cell for *Il17a*^*Cre*^*xTbx21*^*wt*^ (<1000 and >3924 (95th quantile)) and *Il17a*^*Cre*^*xTbx21*^*flox*^ (<1000 and >2467 (95th quantile)) and their percentage of mitochondrial genes (>8%) independently. Additionally, cells with high cell cycle scores (Method: ‘Cell Cycle Scoring’), were removed (*Il17a*^*Cre*^*xTbx21*^*wt*^: S-score >0.2 and G2/M-score > 0.2 / *Il17a*^*Cre*^*xTbx21*^*flox*^: S-score >0.1 and G2/M-score > 0). Next, *Il17a*^*Cre*^*xTbx21*^*wt*^ and *Il17a*^*Cre*^*xTbx21*^*flox*^ were integrated by log-normalization each sample independently (Method: ‘NormalizeData’), selection of the top 500 variable genes common in both samples (Method: ‘Select Integration Features’) and identification of common anchors (Method: ‘Find Integration Anchors’). The samples were integrated using Seurats IntegrateData and the data was scaled by removal of mitochondrial confounding effects (Method: ‘Scale Data’). The PCs 1–20 were used to construct the Shared Nearest Neighbor Graph (Method: ‘Find Neighbours’) and the UMAP-Space (Method: ‘Run UMAP’). The clusters were calculated by the Louvain algorithm (Find Clusters (resolution = 0.5)). Clusters with low alpha/beta TCR expression were removed. Finally, clustering and dimensional reduction steps were performed.

### Differential expression, Modulscores and statistic tests

The differential expressed (DE) genes were determined by using the function ‘FindAllMarkers’ (min.pct = 0.1, logfc.threshold = 0.25, only.pos = T) and subsequently keeping only genes with an adjusted p-value < 0.01. The clusters were annotated according to the DE genes and curated marker genes. We used Seurat’s function ‘AddModulScore’ to build a Th17 score (gene list: *Il17a*, *Il17f*, *Rorc*, *Ccr6*, *Stat3*) and a Th1 score (gene list: *Ifng*, *Tbx21*, *Ccr5*, *Cxcr3*, *Stat4*). The significance between two modul scores were computed using Wilcoxon-test (function: ‘wilcox.test’)

### Th17 trajectory construction and analysis

To construct the trajectories, we ran the R-Package slingshot (version 1.8.0) and its function slingshot (omega = T, stretch = FALSE). Seurat’s UMAP coordinates and clusters were provided as data input. Cluster 3 annotated with Th17 was set as the start cluster. Next, we wanted to determine which genes alter along the slingshot trajectories. We used the R-package trade Seq (version 1.4.0) and fitted a generalized additive model on the genes by applying the method fit GAM (nknots = 6). As count matrix, we took the raw counts of the Seurat object and kept only variable genes. The cell weights and pseudotime was transferred by giving the slingshot object. Subsequently, we tested for genes associated with pseudotime using slingshots association test (lineages = T). To create Fig 4D, we used the method predict Smooth (nPoints = 50) to predict the expression of the top 50 genes ordered by Wald statistic for 50 timestamps along the pseudotime trajectory of lineage 1. Subsequently, we visualized the predicted gene expression of these genes using the function pheatmap (cluster_col = FALSE, border_color = NA, scale = "row”) from the R-Package pheatmap (version 1.0.12).

### Histology

Kidney was fixed overnight in 4% paraformaldehyde, washed with PBS and embedded in paraffin with a tissue processor (Leica, Wetzlar, Germany). For GR1 staining, paraffin blocks were cut with a microtome (Leica) to 1.5 μm sections. Sections were transferred to a kryo frost microscope slide (Super Frost/Plus, Glaswarenfabrik Karl Hecht GmbH& Co. KG, Sondheim vor der Rhön, Germany) and incubated over night at 40°C. Paraffin was removed by a descending alcohol series ended in distillated water (dH_2_O). Sections were framed with Dakopen (DAKO, Hamburg, Germany) to keep solutions on the slide. Slides were unmasked with proteinase digestion, washed with PBS + 0.2% Tween and blocked with blocking buffer from ZytoChem Plus (AP) Polymer Bulk Kit- Polap 100 (Zytomed Systems, Bargteheide, Germany). Slides were incubated with the primary antibody anti-mouse Ly6G (1:5000) NIMP-R14 overnight at 4°C. Biotinylated rabbit anti-rat IgG antibody, mouse adsorbed Vector (1:200) was used as bridge-antibody and incubated at room temperature for 30 min. For AP-reaction incubation with anti-rabbit ap complex from polapkit 30 min at room temperature, and for detection 12 min in new fuchsine solution prepared with 150 mL Tris-sodium puffer (2,08 M Tris, 6,16 M sodium chloride and 32,6 M Tween 20 solved in 1M HCl) supplemented with 7.5 mL 4% sodium nitrite, 0.3 mL Neufuchsin solution (136 mM in 2 M HCl) and 750 mg naphthol-AS-Bi-phosphate mixture (0.044 mmol naphthol-AS-Bi-phosphate and 10.26 mmol NN-dimethylformamide). Before nucleus staining with haematoxylin slides were washed with dH_2_O. Slides were covered with gummi arabicum and cover glass. Slides were analyzed by counting GR1^+^ cells in 10 visual fields and 15 glomeruli in 200x optical magnification (Zeiss Scope.A1 Axio, Oberkochen, Germany). Kidney abscesses per section were counted on these slides.

For CD3 staining, paraffin blocks were cut with a microtome to 1.5 μm sections. Sections were transferred to a kryo frost microscope slide and incubated over night at 40°C. Paraffin was removed by a descending alcohol series ended in dH_2_O. Slides were unmasked by cooking on 90°C in Dako-buffer pH 9 for 15 min and cooled down on room temperature for 25 min. Sections were framed with Dakopen (DAKO, Hamburg, Germany) to keep solutions on the slide. Blocking was performed with blocking buffer from polapkit. The primary antibody rabbit polyclonal anti-human CD3 DAKO (1:1000) was incubated overnight at 4°C. For AP-reaction incubation with anti-rabbit ap complex from polapkit 30 min at room temperature and for detection 12 min in new fuchsin solution in the dark. Before nucleus staining with haematoxylin slides were washed with distillated water. Slides were covered with gummi arabicum and cover glass. Slides were analyzed by counting CD3^+^ cells in 15 visual fields and 15 glomeruli in 400x optical magnification.

For PAS staining, paraffin blocks were cut with a microtome to 1.0 μm sections. Sections were transferred to a microscope slide and incubated over night at 40°C. Paraffin was removed by a descending alcohol series ended in dH_2_O. Sections were incubated for 15 min at room temperature in 1% periodic acid, washed with dH_2_O, incubated in Schiff reagent for 40 min at room temperature and washed with dH_2_O. Nucleus staining was performed by staining with haematoxylin for 1 min and bleached with HCL alcohol. Slides were drained by an ascending alcohol series and were covered in Eukitt and cover glass.

For Gram staining, paraffin blocks were cut with a microtome to 2.0 μm sections. Sections were transferred to a microscope slide and incubated over night at 40°C. Paraffin was removed by a descending alcohol series ended in dH2O. Sections were first incubated 10 min at room temperature in nuclear fast red solution, second in gram´s crystal violet solution for 5 min and third in Lugol’s solution for 1 min at room temperature. Slides were bleaches with Anilin (Emsure, Merck Millipore, Darmstadt, Germany) for 2 min, drained with 100% ethanol and xylol and covered in Eukitt and cover glass.

### Electron microscopy

For electron microscopy the selected part of the mouse kidney was transferred from 4% formaldehyde into a cacodylate buffer with sucrose for 10 min at 80°C. Afterwards, osmium tetroxyde was applied for 2 h. The specimen was washed in cacodylate buffer plus sucrose twice for 5 min. Subsequently, the sample was contrasted with uranyl acetate for 1 h. The specimen was put into ethanol baths with rising ethanol concentrations for 5 min in each bath, followed by bathing in Methyl tert-butyl ether (MTBE) plus epoxide mixture (in a 1:3 dilution) twice for 5 min each. Afterwards, the specimens were embedded in an epoxide mixture at 60°C for 48 h and then at 100°C for 11½ h. Semithin and ultrathin sections were cut on a Leica Microsystems microtome. Grids were purchased from Polyscience. The grids were then analyzed using electron microscopes (EM 109 and EM 902, Zeiss, Oberkochen, Germany) equipped with digital electron microscope cameras (Tröndle). 3 glomeruli from each mouse were analyzed.

### Immunofluorescence microscopy

Indirect immunofluorescence microscopy was performed in 1 μm paraffin-embedded sections of kidneys from *Il17aCre x R26eYFP* mice. Images were captured using a laser confocal microscope (LSM800, Zeiss, Oberkochen, Germany). Primary antibodies against GFP, synaptopodin and endomucin as well as 4′,6-diamidino-2-phenylindole (DAPI) staining (1:10.000) were used.

### Antibodies

The following antibodies were used in this study: CD45PercP/AF700 (30-F11); CD4 BV605/BV650 (RM4-5); CD8 BV785 (53–6.7); CD3 AF700/BV785 (145-2C11); γδTCR BV510/BV605/FITC (eBio GL3); IL17A PE (TC11-18H10), MHC2 BV510 (M5/114.15.2), Ly6C APC-H7 (HK1.4), CD86 BV650 (GL-1), CD80 BV605 (16-10A1), CD127 PE-Cy7 (A7R34), CD90.2 (30-H12), RORγt (Q31-378) and on lineage CD5 (53–7.3), CD11b (M1/70), CD11c (HL-3, BD), CD19 (6D5), CD49b (HMα2), TCR-β (H57-597), GR-1 (RB6-8C5), NK1.1 (PK136) and Ter119 (Ter119) all BioLegend (San Diego, California); CD11b PE-Cy7 (M1/70) and Ly6G PerCP (1A8) all from Life Technologies, Karlsbad, Carlifornia; IFN-γ APC (XMG1.2; eBioscence, Thermo Fisher Scientific, Waltham, Massachusetts); Ly6G (NIMP-R14; Hycult biotech, Uden, The Netherlands); F4/80 APC (BM8, Dianova, Hamburg, Germany); GFP (polyclonal, Abcam, Cambridge, UK); Synaptopodin (polyclonal, Synaptic Systems, Göttingen, Germany); Endomucin (clone V.7C7, Santa Cruz Biotechnology, Dellas, Texas).

### Statistics

Statistical analyses were performed using Graph Pad Prism (La Jolla, CA). Data represent mean ± SEM. The following tests were used: Mann-Whitney test (two-tailed) and Dunnett’s multiple comparison one-way ANOVA analysis. The results are shown as indicated at Figure legends.

## Supporting information

S1 Fig Renal pathology and kidney infiltrating immune cells in S. aureus sepsis. (A) PAS staining of kidney sections from C57BL/6 mice 10 days after *S*. *aureus* infection. (B) Quantification of abscesses in (A) after *S*. *aureus* infection as indicated. (C) CD3^+^ staining of kidney sections 10 days after *S*. *aureus* infection and (D) Quantification of CD3^+^ cells per glomeruli and per hpf as indicated in C57BL/6 mice. (E) GR1^+^ staining of kidney sections 10 days after *S*. *aureus* infection and (F) Quantification of GR1^+^ cells per glomeruli and per hpf as indicated in C57BL/6 mice. (G) Electron microscopy of kidney section from FIR/TIGER/IL-17AKat-w/o-neo mice 10 days after *S*. *aureus* infection. Representative data for one of two independent experiments.
(TIF)

S2 Fig Characterization of lymphocytes during *S*. *aureus* infection. (A) Quantification of renal γδ T cells by flow cytometry from the kidney 10 days after *S*. *aureus* infection. (B and C) Flow cytometry of cytokine production of renal γδ T cells of C57BL/6 mice 10 days after *S*. *aureus* infection (*p<0.05, unpaired t-test, two-tailed, representative for one of three independent experiments). (D) Quantification of renal ILCs by flow cytometry from the kidney 10 days after *S*. *aureus* infection. (E) Flow cytometry and (F) quantification of cytokine production of renal ILCs 10 days after *S*. *aureus* infection (*p<0.05, representative for one of two independent experiments). (G) Quantification of renal CD11b^+^ cells at day 10 after *S*. *aureus* infection. (H) Flow cytometry and (I) quantification of renal CD11b^+^ cells of at day 10 after *S*. *aureus* infection. (J) Flow cytometry and (K) quantification of CD11b^+^ cells from the liver at day 10 after *S*. *aureus* infection. (L-N) Flow cytometry of hepatic CD11b^+^ cells at day 10 after *S*. *aureus* infection. Bars representing mean, individual mice displayed by dots.
(TIF)

S3 Fig Conversion of Th17 cells Th1-like but not regulatory phenotypes in *S*. *aureus* sepsis. (A) Flow cytometry of renal and intestinal Th17 fate cells and Tr1exTh17 cells (IL-17Kat^neg^FoxP3^neg^YFP^+^IL10eGFP^+^; gated on ex Th17) of Fate^+^ mice after *S*. *aureus* infection as indicated (SILP: small intestine lamina propria; bars representing mean, individual mice displayed by dots. (B) Slingshot trajectory analysis of renal Th17 cells (cluster 3) from *Il17aCre x R26eYFP* mice (n = 5) 10 days after *S*. *aureus* infection into different cell states (related to Fig 4). (C) Trajectories of CD4^+^YFP^+^ cells from *Il17aCre x R26eYFP* x *Tbx21-flox* mice (n = 6) 10 days after *S*. *aureus* infection into different cell states (related to Fig 5).
(TIF)

S4 Fig Tbx21 expression in renal non-Th17 cells. (A) Flow cytometry of renal YFP negative CD4^+^ T cells 10 days after *S*. *aureus* infection as indicated and (B) Quantification of cytokine expression; bars representing mean, individual mice displayed by dots, not significant (n.s.), in Dunnett’s multiple comparison one-way ANOVA analysis (representative data for one of two independent experiments). (C and D) Flow cytometry of renal YFP^+^ CD4^+^ T cells at day 10 after *S*. *aureus* infection and anti-IFN-γ antibody (** *p*<0.01, *** *p*<0.001 in Dunnett’s multiple comparison one-way ANOVA analysis). (E) Flow cytometry and (F) quantification of cytokine producing of YFP positive γδ-T cells; dots representing mean ± SEM (each time point represents the data of n = 4–5, representative of one from two independent experiments). (G) Flow cytometry and (H) quantification of renal CD11b^+^ cells from *Rag1*^-/-^ m+ice. Bars representing mean, individual mice displayed by dots.
(TIF)
