# Supplementary figures and images for: Th17 cell plasticity towards a T-bet-dependent Th1 phenotype is required for bacterial control in Staphylococcus aureus infection
Source: PLoS Pathog. 2022 Apr 21;18(4):e1010430. doi: 10.1371/journal.ppat.1010430 (PMC9064098; doi:10.1371/journal.ppat.1010430)

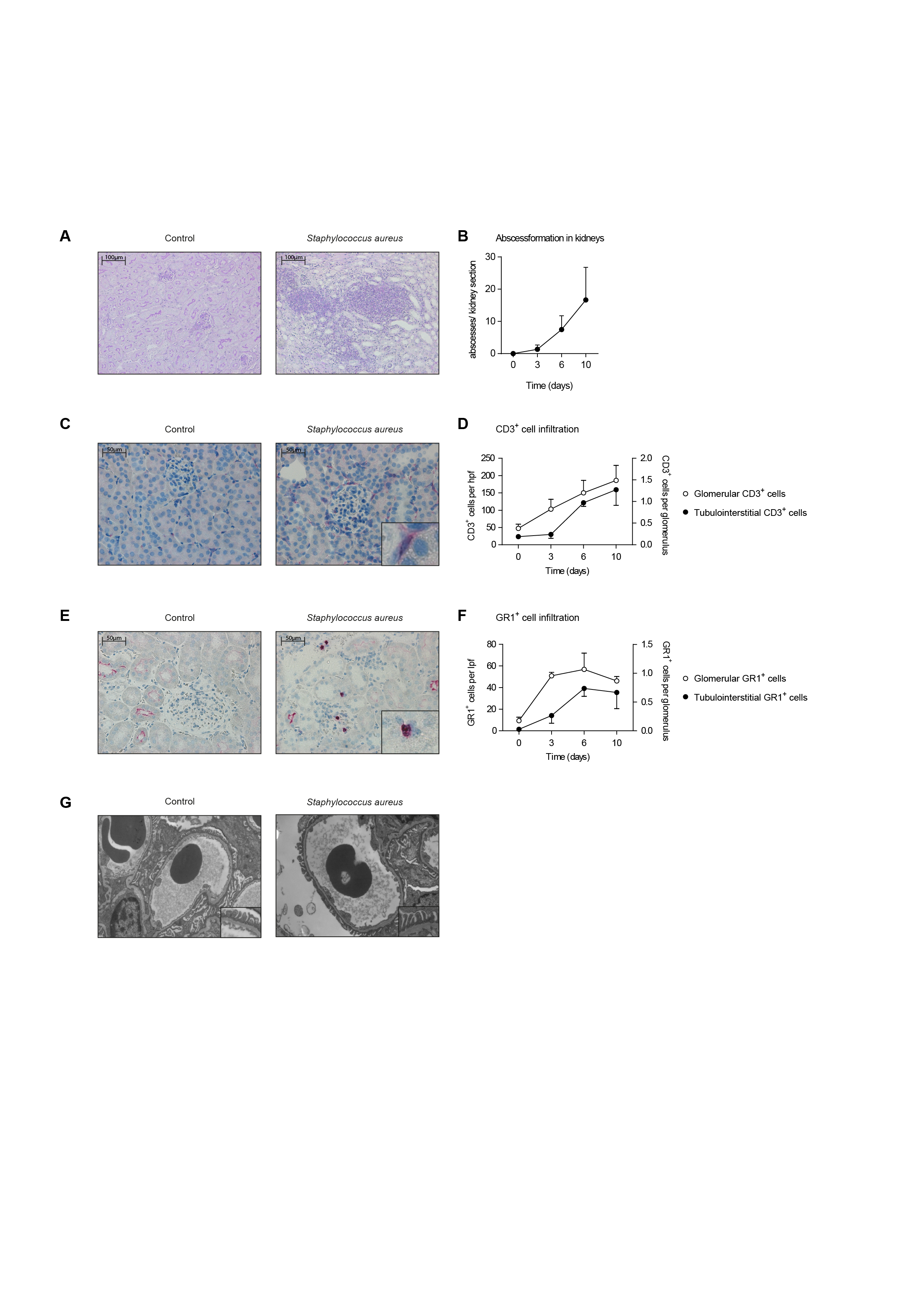

Supplement: S1 Fig — (A) PAS staining of kidney sections from C57BL/6 mice 10 days after S. aureus infection. (B) Quantification of abscesses in (A) after S. aureus infection as indicated. (C) CD3+ staining of kidney sections 10 days after S. aureus infection and (D) Quantification of CD3+ cells per glomeruli and per hpf as indicated in C57BL/6 mice. (E) GR1+ staining of kidney sections 10 days after S. aureus infection and (F) Quantification of GR1+ cells per glomeruli and per hpf as indicated in C57BL/6 mice. (G) Electron microscopy of kidney section from FIR/TIGER/IL-17AKat-w/o-neo mice 10 days after S. aureus infection. Representative data for one of two independent experiments. (TIF) [file ppat.1010430.s001.tif]

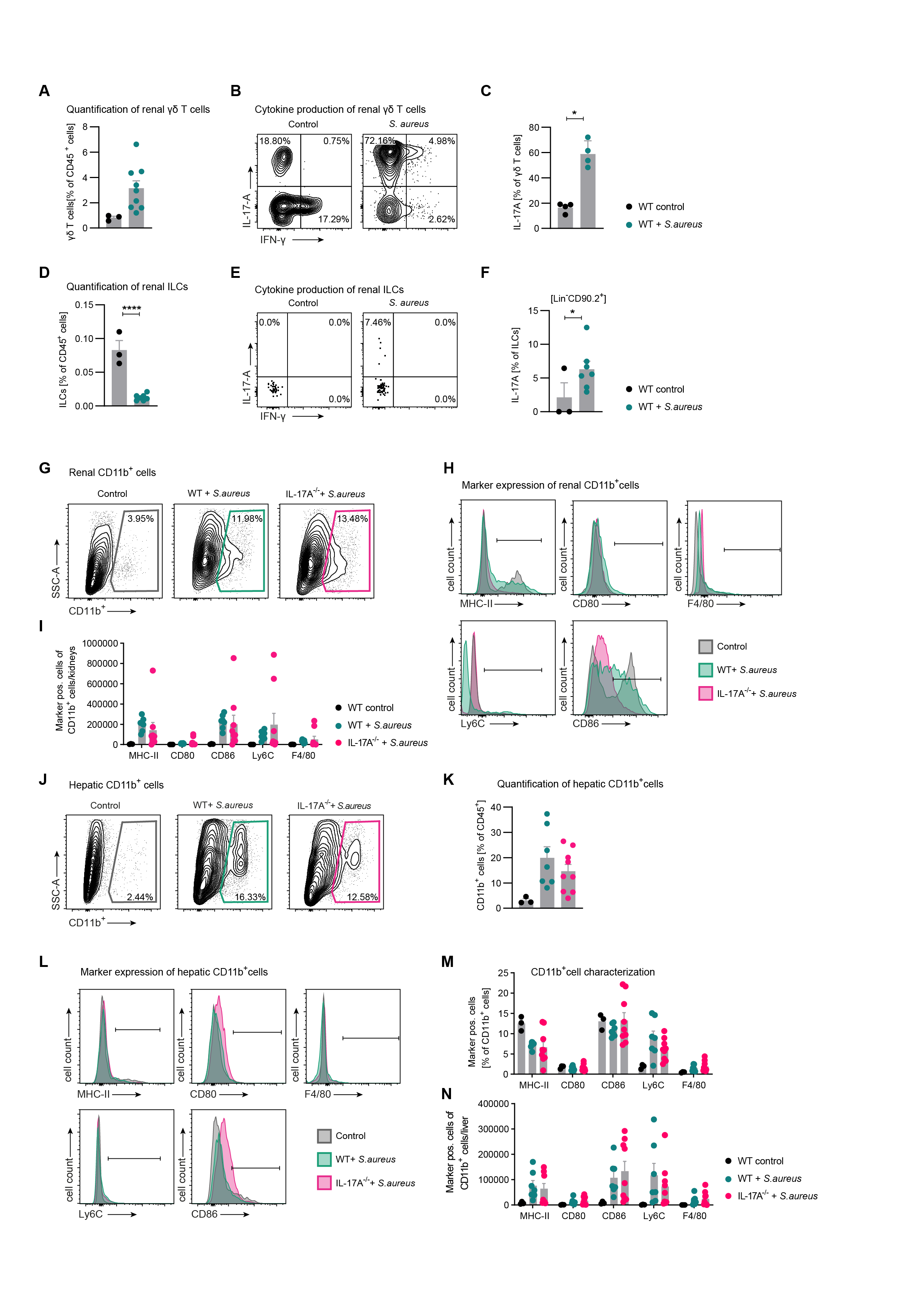

Supplement: S2 Fig — (A) Quantification of renal γδ T cells by flow cytometry from the kidney 10 days after S. aureus infection. (B and C) Flow cytometry of cytokine production of renal γδ T cells of C57BL/6 mice 10 days after S. aureus infection (*p<0.05, unpaired t-test, two-tailed, representative for one of three independent experiments). (D) Quantification of renal ILCs by flow cytometry from the kidney 10 days after S. aureus infection. (E) Flow cytometry and (F) quantification of cytokine production of renal ILCs 10 days after S. aureus infection (*p<0.05, representative for one of two independent experiments). (G) Quantification of renal CD11b+ cells at day 10 after S. aureus infection. (H) Flow cytometry and (I) quantification of renal CD11b+ cells of at day 10 after S. aureus infection. (J) Flow cytometry and (K) quantification of CD11b+ cells from the liver at day 10 after S. aureus infection. (L-N) Flow cytometry of hepatic CD11b+ cells at day 10 after S. aureus infection. Bars representing mean, individual mice displayed by dots. (TIF) [file ppat.1010430.s002.tif]

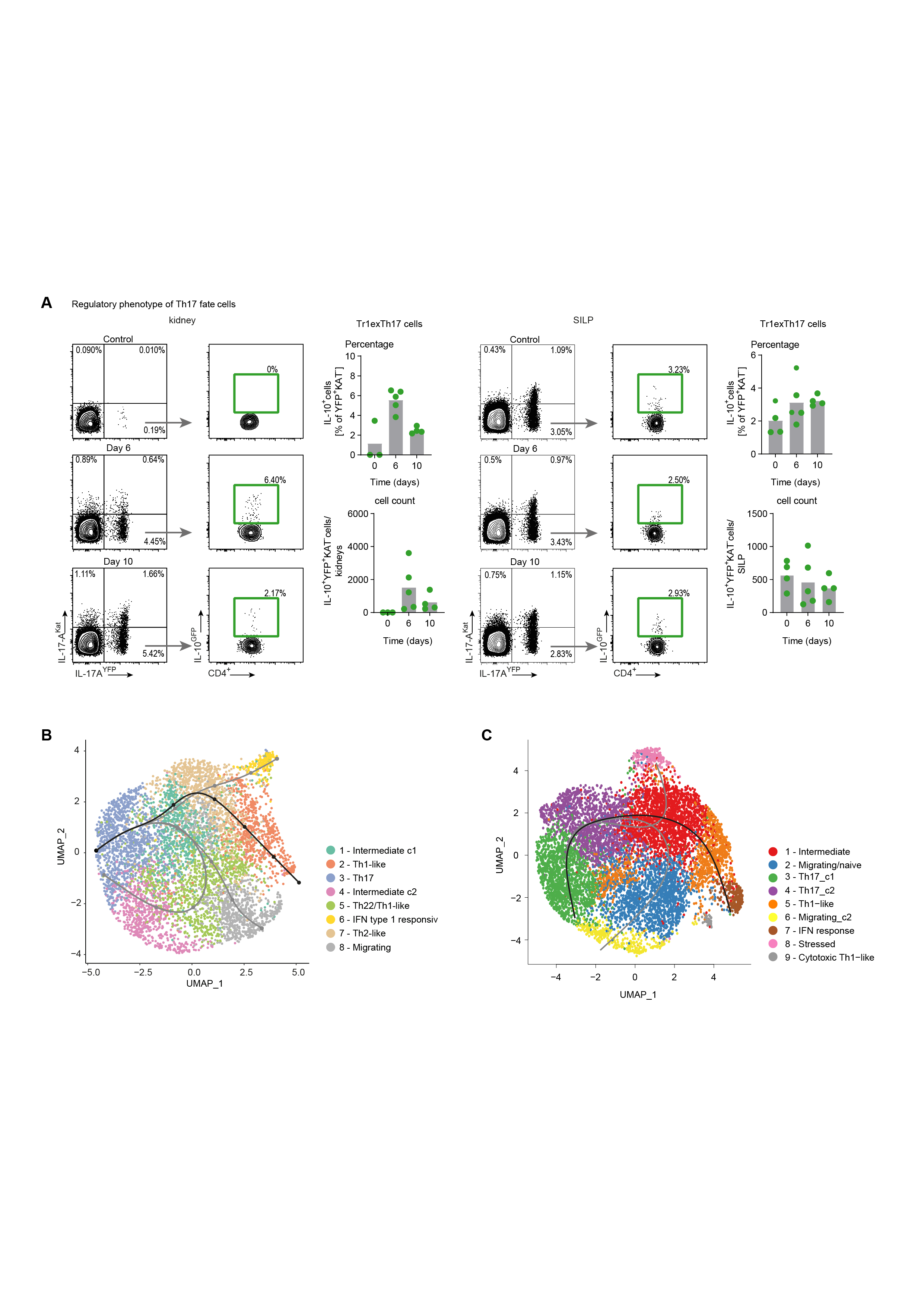

Supplement: S3 Fig — (A) Flow cytometry of renal and intestinal Th17 fate cells and Tr1exTh17 cells (IL-17KatnegFoxP3negYFP+IL10eGFP+; gated on ex Th17) of Fate+ mice after S. aureus infection as indicated (SILP: small intestine lamina propria; bars representing mean, individual mice displayed by dots. (B) Slingshot trajectory analysis of renal Th17 cells (cluster 3) from Il17aCre x R26eYFP mice (n = 5) 10 days after S. aureus infection into different cell states (related to Fig 4). (C) Trajectories of CD4+YFP+ cells from Il17aCre x R26eYFP x Tbx21-flox mice (n = 6) 10 days after S. aureus infection into different cell states (related to Fig 5). (TIF) [file ppat.1010430.s003.tif]

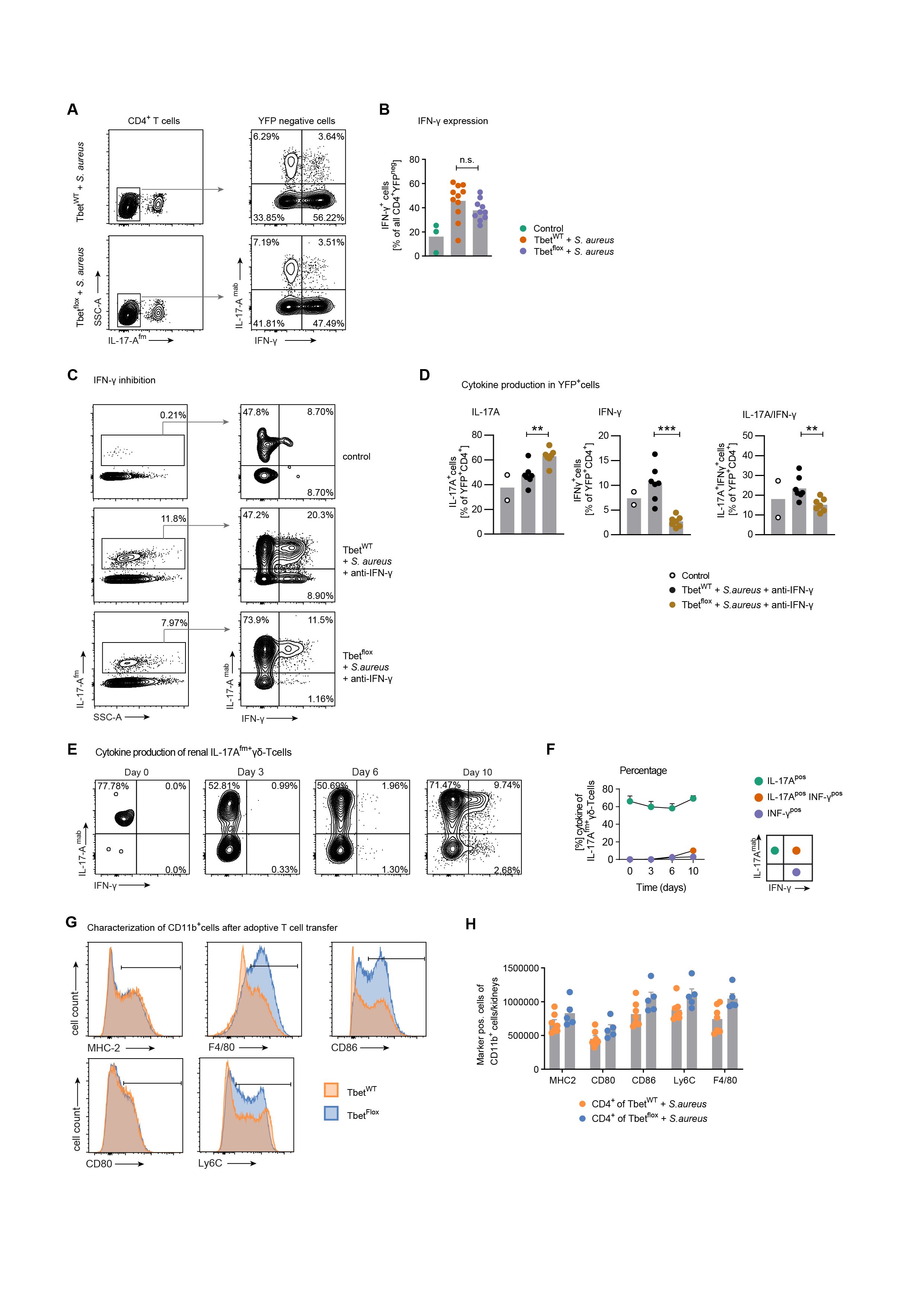

Supplement: S4 Fig — (A) Flow cytometry of renal YFP negative CD4+ T cells 10 days after S. aureus infection as indicated and (B) Quantification of cytokine expression; bars representing mean, individual mice displayed by dots, not significant (n.s.), in Dunnett’s multiple comparison one-way ANOVA analysis (representative data for one of two independent experiments). (C and D) Flow cytometry of renal YFP+ CD4+ T cells at day 10 after S. aureus infection and anti-IFN-γ antibody (** p<0.01, *** p<0.001 in Dunnett’s multiple comparison one-way ANOVA analysis). (E) Flow cytometry and (F) quantification of cytokine producing of YFP positive γδ-T cells; dots representing mean ± SEM (each time point represents the data of n = 4–5, representative of one from two independent experiments). (G) Flow cytometry and (H) quantification of renal CD11b+ cells from Rag1-/- m+ice. Bars representing mean, individual mice displayed by dots. (TIF) [file ppat.1010430.s004.tif]
